# Concurrence of immune thrombocytopenic purpura and thrombotic thrombocytopenic purpura: a case report and review of the literature

**DOI:** 10.1186/s13256-023-03762-y

**Published:** 2023-02-08

**Authors:** Hung-Chen Lin, Jinxiang Huang, Jing Huang, Li-Jun Zhang, Xiao-Wu Yin, Jian-Cheng Yang, Xiao-Yan Huang

**Affiliations:** 1 Division of Cardiology, Department of Medicine, The University of Hong Kong-Shenzhen Hospital, Shenzhen, China; 2 Department of Medicine, The University of Hong Kong-Shenzhen Hospital, Shenzhen, China; 3 Division of Hematology, Department of Medicine, The University of Hong Kong-Shenzhen Hospital, Shenzhen, China; 4 Division of Rheumatology, Department of Medicine, The University of Hong Kong-Shenzhen Hospital, Shenzhen, China; 5 Department of Pathology, The University of Hong Kong-Shenzhen Hospital, Shenzhen, China

**Keywords:** Immune thrombocytopenia purpura, Thrombotic thrombocytopenia purpura, Vaccine-induced thrombotic thrombocytopenia

## Abstract

**Background:**

Immune thrombocytopenic purpura and thrombotic thrombocytopenic purpura are both causes of thrombocytopenia. Recognizing thrombotic thrombocytopenic purpura is crucial for subsequent treatment and prognosis. In clinical practice, corticosteroids and rituximab can be used to treat both immune thrombocytopenic purpura and thrombotic thrombocytopenic purpura; plasma exchange therapy is the first-line treatment in thrombotic thrombocytopenic purpura, while corticosteroids are strongly recommended as first-line treatment in immune thrombocytopenic purpura. The differential diagnosis of immune thrombocytopenic purpura and thrombotic thrombocytopenic purpura is essential in clinical practice. However, case reports have suggested that immune thrombocytopenic purpura and thrombotic thrombocytopenic purpura can occur concurrently.

**Case presentation:**

We report the case of a 32-year-old Asian female without previous disease who presented with pancytopenia, concurrent with immune thrombocytopenic purpura and thrombotic thrombocytopenic purpura. The morphology of the megakaryocytes in the bone marrow indicated immune-mediated thrombocytopenia. The patient received glucocorticoid treatment, and her platelet count increased; however, schistocytes remained high during the course of the therapy. Further investigations revealed ADAMTS13 activity deficiency and positive ADAMTS13 antibodies. The high titer of antinuclear antibody and positive anti-U1-ribonucleoprotein/Smith antibody indicated a potential autoimmune disease. However, the patient did not fulfill the current criteria for systemic lupus erythematosus or mixed connective tissue disease. The patient responded well to plasma exchange therapy, and her platelet count remained normal on further follow-up.

**Conclusions:**

Concurrence of immune thrombocytopenic purpura and thrombotic thrombocytopenic purpura is rare, but clinicians should be aware of this entity to ensure prompt medical intervention. Most of the reported cases involve young women. Human immunodeficiency virus infection, pregnancy, and autoimmune disease are the most common underlying conditions.

## Introduction

Immune thrombocytopenic purpura (ITP) and thrombotic thrombocytopenic purpura (TTP) are distinct diseases that cause thrombocytopenia. ITP is defined as a platelet count below 100,000 platelets per cubic millimeter, excluding known causes of thrombocytopenia; in addition, platelet-associated immunoglobulin (Ig) M or IgG is commonly identified [1]. Primary ITP accounts for 80% of cases; the additional 20% are secondary to some other disease, such as infection, autoimmune disease, malignancy, or primary immune deficiency [2]. TTP is caused by severe deficiency of the von Willebrand factor (vWF)-cleaving metalloproteinase, ADAMTS13, which leads to the formation of platelet-rich thrombi in the vasculature [3]. TTP is a rare and life-threatening disease with an average annual prevalence of 10 cases/1 million people and a mortality rate of 10–20% [4]. Recognizing TTP is crucial for timely treatment and prognosis. In clinical practice, although corticosteroids and rituximab can be used to treat both ITP and TTP, plasma exchange therapy (PEX) is the first-line treatment for TTP; in contrast, corticosteroids are strongly recommended as first-line treatment in ITP [5, 6]. Therefore, differential diagnosis of ITP and TTP is essential in clinical practice. Concurrence is rare; however, previous case reports have shown that ITP and TTP can occur concurrently in acquired immune deficiency syndrome (AIDS), pregnancy, and Sjögren’s syndrome [7–9]. We report herein a case of concurrent ITP and TTP in a patient without knowledge of any underlying diseases.

## Methods

### Case report

In this case report, we analyze the patient’s clinical presentation, laboratory results, treatment, and outcome.

### Literature review and data extraction

The MEDLINE database was used to search for all published literature on ITP complicating TTP. The Medical Subject Headings (MESH) terms we used were (((idiopathic thrombocytopenic purpura [Mesh Terms]) OR (immune thrombocytopenia [Mesh Terms])) AND (thrombotic thrombocytopenic purpura [Mesh Terms])) OR (immune thrombotic thrombocytopenic purpura [Mesh Terms]). There were no restrictions on article type or publication date, and 497 results were obtained. After removal of duplicated articles and articles without access to full-text reports, 16 case reports in any language, on patients of any age or race diagnosed with ITP and TTP were included. The following data retrieved from each report are presented in Table 1: (1) first author's name and year of publication, (2) age and sex of the patient, (3) underlying conditions, (4) significant laboratory characteristics, (5) ADAMTS13 activity and antibodies, if reported, (6) main clinical presentations, (7) therapy, and (8) patient outcomes. The search was conducted on 6 June 2022.

**Table 1:** Summary of cases of immune thrombocytopenic purpura complicated with thrombotic thrombocytopenic purpura

Reference	Underlying condition	Age and sex	Race	PLT (× 10^9^)	LDH (U/L)	ADAMTS13 activity	ADAMTS13 inhibitor	Sequence	Clinical presentation	Therapy	Outcome
Zacharski 1976 [12]	None	26/M	NS	50	NS	NS	NS	ITP Prior to TTP	Fever, lethargy, dizziness, weakness of extremities, aphasia	St, SP, aspirin, dipyridamole	R
Stein 1984 [13]	None	22/M	White	25	NS	NS	NS	TTP prior to ITP	Fever, ecchymosis, seizure, unresponsive, AKI	St, PEX, SP	R
Stein 1984 [13]	None	34/F	NS	12	NS	NS	NS	TTP prior to ITP	Fever, ecchymosis, confusion, headache	St, PEX, SP, V	R
Stein 1984 [13]	Postpartum	17/F	Black	12	NS	NS	NS	TTP prior to ITP	Fever, AKI, nerve palsy	St, PEX, SP	R
Meiscnberg 1988 [14]	HIV	25/M	NS	10	945	NS	NS	TTP prior to ITP	Nausea, vomiting, jaundice	PEX	R
Krupsky 1991 [15]	None	14/F	Indian	41	NS	NS	NS	ITP prior to TTP	Fever, headache, vaginal bleeding, vomiting, aphasia, paresis, seizure, cardiac arrest	St, SP, V, PEX	Died
Routy 1991 [16]	HIV	35/M	NS	40	470	NS	NS	ITP prior to TTP	Fever, confusion, RF	St, PEX, SP	Died
Routy 1991 [16]	HIV	31/M	NS	15	27.5	NS	NS	ITP prior to TTP	Fever, nausea, icterus, convulsion, AKI	PEX, V, St	R
Shivaram 1992 [17]	HIV	34/M	NS	10	NS	NS	NS	ITP prior to TTP	Fever, mental status changes	PEX	R
Olenic 1994 [18]	Postpartum	15/F	NS	7	1932	NS	NS	ITP prior to TTP	Ecchymosis	St, intravenous IG, PEX	R
Yospur 1996 [7]	HIV	42/M	NS	NS	NS	NS	NS	ITP prior to TTP	Fever, mental status changes	PEX, St, V	R But died of PJP
Yospur 1996 [7]	HIV	38/F	Hispanic	7	778	NS	NS	Concurrence	Headache, ecchymosis, petechiae, aphasia, paresthesias, cramping, seizure	St, PEX	R
Prasad 1996 [19]	HIV	9/M	NS	6	1569	NS	NS	ITP prior to TTP	Fever, vomiting, petechiae, paresthesia, aphasia, hemiparesis	St, V, intravenous IG, PEX	R
Baron 2001 [20]	SLE/MCTD	55/F	African American	14	1107	NS	NS	TTP Prior to ITP	Fever, seizures, unresponsive, AMI, AKI, ecchymosis	St, PEX, intravenous IG	R
Baron 2001 [20]	RA	62/F	White	14	2656	NS	NS	ITP prior to TTP	Petechiae, cerebral ischemia	St, V, PEX and intravenous IG	R
Baron 2001 [20]	Positive Coombs test	28/F	African American	13	1204	NS	NS	TTP prior to ITP	Fever, ecchymosis, headache, CPA	PEX, St	Died
Baron 2001 [20]	None	45/F	Caucasian	14	normal	NS	NS	TTP prior to ITP	Fever, petechiae, mental status changes, hematuria	PEX, St, intravenous IG	R
Bayrakta 2010 [20]	Metastatic neuroendocrine tumor on bevacizumab	63/F	Hispanic	16	4233	normal	NS	Concurrence	Pallor, jaundice	PEX, intravenous IG, V, St, rituximab, CTX	R
Changela 2011 [22]	Prostate cancer	60/M	NS	8	1348	12%	( +)	ITP prior to TTP	Fever gingival bleed, chest pain, shortness of breath, hematuria, intracranial bleed, AKI	St, PEX, intravenous IG, rituximab	R
Farhat 2012 [23]	None	42/F	African American	34	NS	NS	NS	TTP prior to ITP	Slurring of speech, left upper extremity weakness	St, intravenous IG, V, PEX	R
Husban 2018 [8]	Postpartum	30/F	Middle Eastern	56	28.4	11%	NS	ITP prior to TTP	Fever ecchymosis, headache, fatigue, dizziness liver function impairment	St, PEX, MMF, rituximab	R
Devon 2021 [9]	Primary Sjögren’s syndrome	72/M	Caucasian	9	2,399	9%	( +)	ITP prior to TTP	Ecchymosis, epistaxis, hematuria, AKI	St, MMF, romiplostim, intravenous IG, PEX, rituximab	R
Hangping Ge [24] 2022	None	33/F	Asian	5	543	0%	( +)	ITP prior to TTP	Ecchymosis, weakness, heavy menstrual bleeding	St, intravenous IG, PEX, rituximab, MMF	R
Present study	None	32/F	Asian	28	330	1.665%	( +)	Concurrence	Petechiae, headache	St, PEX	R

*PLT* platelet, *NS* not specified, *ITP* immune thrombocytopenic purpura, *TTP* thrombocytopenic purpura, *St* steroids, *SP* splenectomy, *R* remission, *IVIG* intravenous immunoglobulin therapy, *AKI* acute kidney injury, *RF* renal failure, *PEX* plasma exchange, *V* Vincristine, *AMI* acute Myocardial infarction, *CPA* cardiorespiratory arrest, *CTX* cyclophosphamide, *PJP* pneumocystis Jiroveci pneumonia, *SLE* systemic lupus erythematosus, *MCTD* mixed connective tissue disease, *RA* rheumatoid arthritis, *MMF* mycophenolate mofetil

## Results

### Case report

A 32-year-old Asian female presented to the University of Hong Kong-Shenzhen Hospital on 6 November 2021, complaining of a headache for 6 months and purpura for 3 weeks. The patient had been experiencing intermittent headaches with tinnitus for 6 months, prior to admission. Complete blood cell count (CBC) revealed a white blood cell (WBC) count of 3.07 × 10^9^/L, hemoglobin (HGB) of 10^6^ g/L, and platelet (PLT) count of 210 × 10^9^/L. Brain magnetic resonance imaging (MRI) showed normal findings.

Three weeks prior to admission, the patient noticed purpura on her extremities, and she also complained of increased menstrual flow. She had received two doses of the inactivated coronavirus disease 2019 (COVID-19) vaccine in May 2021 and in June 2021. She had no history of significant medical illness, except for one stillbirth. She denied alcohol, tobacco, or drug abuse. She had never received a transfusion. She took no medications on admission, and there was no significant family medical history.

Physical examination revealed an afebrile, normotensive female. There were no hemorrhage lesions on her palate; however, multiple ecchymoses and petechiae were observed over the body. There was mild tenderness in the upper limb muscles. There was no splenomegaly, lymph node hypertrophy, or liver hypertrophy. Her physical examination was otherwise unremarkable. Chest computed tomography revealed ground-glass nodules in the left upper lung and solid nodules in the left lower lung, which were considered benign lymph nodes by radiologists. Brain MRI revealed a choroid plexus cyst. There were no significant findings from abdominal ultrasound and echocardiogram.

The urine pregnancy test was negative. CBC revealed a WBC count of 3.41 × 10^9^/L, HGB of 77 g/L, PLT count of 28 × 10^9^/L, mean corpuscular volume (MCV) of 96.6 fL, and vitamin B12 level of 319 pg/mL (reference: 180–914 pg/mL). Blood smear showed normal leukocyte morphology, the reticulocyte percentage was 6%, and the absolute reticulocyte count was 0.094 × 10^12^/L. The serum lactate dehydrogenase level was 330 U/L. The percentage of CD55^−^ erythrocytes and CD59^−^ erythrocytes of the total erythrocytes was less than 0.5% each. The level of glucose-6-phosphate was 23 U/L, which was normal. Thyroid function was normal, with a slightly elevated thyroglobulin antibody titer (110.3 U/mL [reference: 0–60 U/mL]). d-dimer was 1.52 µg/mL, and haptoglobin was < 25 mg/dL. Immune function test revealed IgG of 17.13 g/L complement protein 3 (C3) of 0.63 g/L. The anti-nuclear antibody (ANA) test was positive at a titer > 1:1000, with a speckled pattern. The extractable nuclear antibody profile showed strong positivity (3+) for anti-ribonucleoprotein/Smith (U1RNP/Sm) antibody. The direct Coombs’ test, anti-double-stranded deoxyribonucleic acid (dsDNA) antibodies, antineutrophil cytoplasmic antibodies, anticardiolipin antibodies, anti-red blood cell antibodies, lupus anticoagulants, antiplatelet antibodies, and anti-β2-glycoprotein antibodies were negative. Coagulation, liver function (including bilirubin), kidney function, electrolytes, erythrocyte sedimentation rate, C-reactive protein, and iron studies were all within normal limits. Tumor markers were all negative. The urinalysis was unremarkable.

**Fig. 1:**
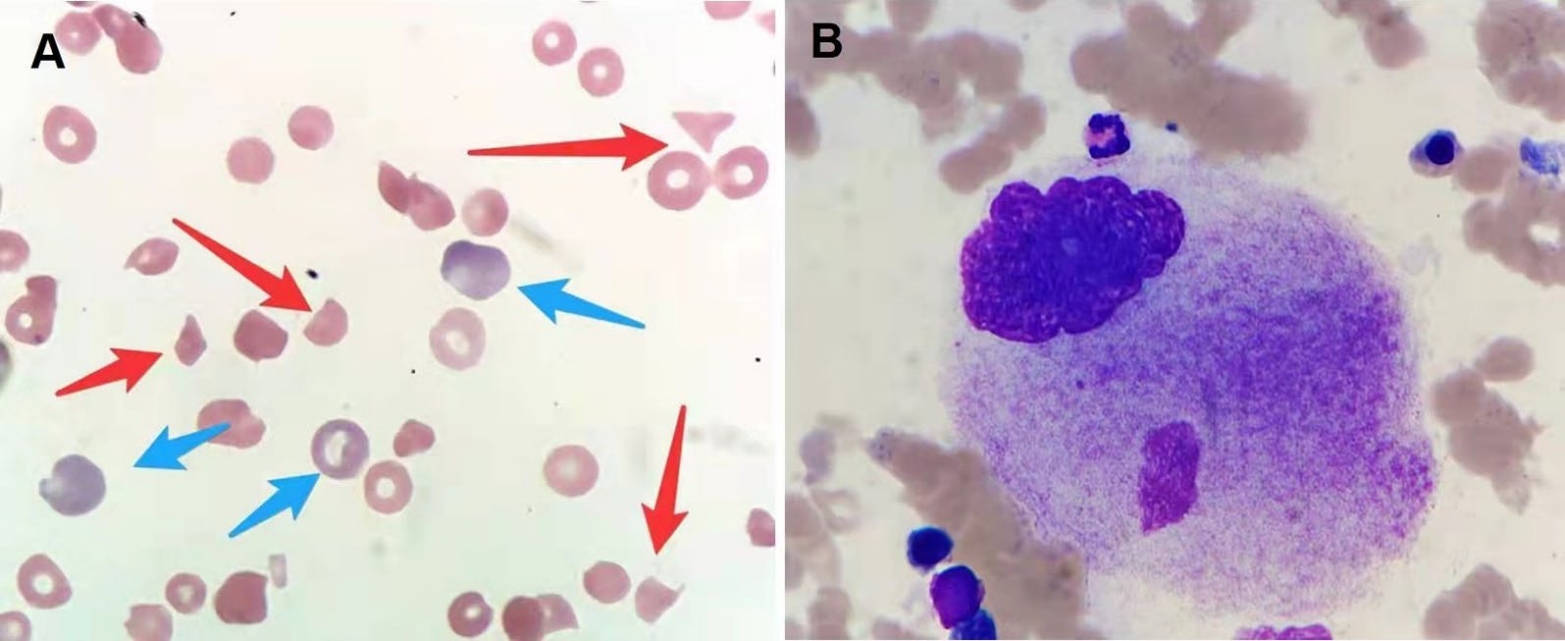
**A** The peripheral blood smear shows schistocytes (red arrows) and reticulocytes (blue arrows) (×400). **B** Bone marrow megakaryocyte with increased size and platelet production deficiency (×1000)

The peripheral blood smear revealed many fragmented red blood cells with increased polychromasia and decreased platelets (Fig. 1A). Bone marrow biopsy revealed relative erythroid hyperplasia and a normal megakaryocyte count (Fig. 1B). The investigations for pathogens, including human immunodeficiency virus (HIV), hepatitis B virus, hepatitis C virus, respiratory viruses, Epstein–Barr virus, cytomegalovirus, and *Helicobacter pylori*, were negative. The clinical scenario revealed Coombs’-negative hemolytic anemia, complicated with immune-mediated thrombocytopenia that was possibly caused by an autoimmune disease.

The patient was initially treated with intravenous methylprednisolone 40 mg for 1 day, but the schistocytes in the peripheral blood increased to over 6%. The methylprednisolone dose was then increased to 240 mg for 5 days. The patient’s platelet count increased to 216 × 10^9^/L, but the schistocytes remained at 5%. The PLASMIC score was high. The PLASMIC score is a seven-component clinical prediction tool that was developed to reliably assess the pretest probability of severe ADAMTS13 deficiency [*C* statistic 0.96, 95% confidence interval (CI) 0.92–0.98] [9, 10]. TTP was suspected. The ADAMTS13 activity was 1.65%, and the ADAMTS13 inhibitor assay was positive; therefore, a diagnosis of TTP was considered. The patient then received six cycles of PEX. Cyclosporine was added, and the dosage was titrated to 150 mg per day. Following treatment, the PLT count remained normal, schistocytes were reduced to 1–2%, ADAMTS13 activity increased to 63.99%, and anti-ADAMTS13 antibody was negative. During hospitalization, the patient did not present any signs of thrombosis, and all blood vessel ultrasounds were normal. She was discharged and managed in an outpatient clinic with tapered oral methylprednisolone. At the last visit, on 2 June 2022, oral methylprednisolone and cyclosporine were discontinued, and the patient only took hydroxychloroquine 0.2 g once daily. The patient’s CBC remained normal.

### Systemic literature review

Sixteen publications [7–9, 11–23] were evaluated in detail, and a total of 24 patients (including our patient) were included in this qualitative analysis. The patients’ ages ranged from 9 to 72 years old, and the mean age was 36 years. There were 14 females and 10 males; 7 patients were HIV positive, 3 patients were postpartum women, 3 had autoimmune diseases (such as systemic lupus erythematosus (SLE), rheumatoid arthritis, and Sjögren’s syndrome), 1 case was drug related, 1 patient had a history of a tumor, 1 had a positive Coombs’ test, and the remaining 8 patients had no known underlying medical conditions. The common laboratory characteristics were thrombocytopenia, elevated schistocytes, and elevated lactate dehydrogenase (LDH). Only 6 patients’ ADAMTS13 activity was detected (ranging from 0% to normal). The most common symptoms were mucocutaneous bleeding (*n* = 16), neurological symptoms (*n* = 16), and fever (*n* = 14). All patients received PEX and/or steroids. Intravenous immunoglobulin, splenectomy, vincristine, and rituximab were the most common assistant therapies. Following treatment, 20 patients were in remission, and 4 patients (16.7%) died. The results are presented in Table 1.

## Discussion

We present a case of thrombocytopenia in a previously healthy woman. The morphology of megakaryocytes in the bone marrow indicated immune-mediated thrombocytopenia; furthermore, the platelet count responded well to glucocorticoids. The patient also presented with anemia, with slightly increased MCV, increased reticulocyte level, decreased haptoglobin, and increased lactate dehydrogenase (LDH) level, indicating hemolysis. The patient presented with a high level of ANA and positive anti-U1-RNP, which indicated potential autoimmune hemolysis; however, the negative Coombs’ test and negative anti-red blood cell antibodies made autoimmune hemolysis less likely. Furthermore, the patient did not exhibit other clinical signs to fulfill the current criteria for systemic lupus erythematosus (SLE) or mixed connective tissue disease. The schistocytes remained high during glucocorticoid therapy, which indicated intravascular hemolysis, and further investigations revealed ADAMTS13 activity deficiency and positive ADAMTS13 antibody, which confirmed the diagnosis of TTP.

During the COVID-19 pandemic, new vaccines were developed without full clinical trials. Some vaccines have been reported to be associated with *de novo* ITP, ITP exacerbation, and vaccine-induced immune thrombotic thrombocytopenia (VITT), especially the adenoviral vector-based vaccine (ChAdOx1 nCov-19, Ad26.COV2.S) [24]. VITT is caused by antibodies that recognize platelet factor 4 (PF4) bound to platelets [25]. The pathomechanism of VITT is under investigation; current studies suggest a two-hit process in which the vaccine stimulates neoantigen formation (first hit), in addition to a systemic inflammatory response (second hit), which together lead to the production of anti-PF4 antibodies [26]. Anti-PF4 antibodies lead to the inhibition of ADAMTS13 activity, which is unable to regulate the multimeric size of vWF [27]. Ultra-sized vWF multimers can accumulate in the plasma, leading to the formation of platelet-rich microthrombi [28]. VITT likely develops in a narrow window, 5–10 days post-vaccination, leading to the identification of cases typically between 5 and 30 days post vaccination [29]. A case of possible VITT related to the inactivated COVID-19 vaccine was published. In this case, the symptoms occurred 2 weeks after vaccination [30]. Our patient received the inactivated COVID-19 vaccine 5 months prior to the onset of symptoms, which made a diagnosis of VITT less likely.

Furthermore, our patient presented with hemolytic anemia and thrombocytopenia, making Evans syndrome a possible differential diagnosis; however, the negative direct Coombs’ test, and the high percentage of schistocytes, made this diagnosis less likely. Therefore, the case was diagnosed as concurrence of ITP and TTP.

TTP is a rare form of thrombotic microangiopathy (TMA), characterized by microangiopathic hemolytic anemia (MAHA), severe thrombocytopenia, and ischemic end-organ damage resulting from the formation of platelet-rich thrombi in the microvasculature [31]. PEX is the primary treatment for TTP, whereas glucocorticoids have been routinely added as an assisted therapy alongside PEX [32]. Glucocorticoids are thought to hasten recovery by reducing the production of ADAMTS13 inhibitors, and reducing cytokine production and autoantibody-mediated clearance of ADAMTS13. According to the 2020 guidelines of the International Society on Thrombosis and Hemostasis (ISTH), glucocorticoids are recommended, in addition to PEX, for initial treatment of TTP, despite the lack of randomized trials on this combination therapy [32]. However, the efficacy of monotherapy with glucocorticoids in TTP has been demonstrated in observational studies. In research on 54 patients with TTP treated with corticosteroids alone, 24 patients (44.4%) did not respond to the treatment [33]. In contrast, another study comparing prednisone versus cyclosporin found that prednisone was superior to cyclosporin for increasing ADAMTS13 activity, and suppressing anti-ADAMTS13 antibodies [34]. The efficacy of glucocorticoid monotherapy in TTP is varied; however, glucocorticoid therapy is fundamental to ITP treatment, and patients with ITP usually respond well to glucocorticoid therapy [35]. Rituximab, a monoclonal antibody directed against the B-cell lineage-specific CD20 antigen, can be added to corticosteroid and PEX therapy to increase the response rate in refractory TTP [36]. The newest TTP therapy is caplacizumab, a humanized, single-variable-domain, anti-vWF immunoglobulin that targets the A1 domain of vWF [37], preventing interaction with the platelet glycoprotein Ib-IX-V receptor. Caplacizumab showed value when added to the standard treatment for acquired TTP; it shortens the time to normalize platelets and decreases the recurrence rate [38].

Sequential or concurrent ITP and TTP have been reported in the literature. We reviewed 23 sequential, or concurrent, ITP and TTP cases (Table 1). Based on these case reports, patients with concurrent or sequential ITP and TTP usually present with microangiopathy; however, our case lacked the features of microangiopathy . This could be attributed to the low platelet count in the early stage of ITP, which led to the formation of fewer platelet-rich thrombi. Further investigation is needed to confirm this hypothesis.

This is a rare case of concurrent ITP and TTP in a previously healthy woman. This case highlights the importance of considering all possible causes of thrombocytopenia, especially when specific treatments (i.e., PEX, glucocorticoids) should be given in the first line.

## Conclusion

Concurrence of ITP and TTP is rare; however, clinicians should be aware of this entity to ensure prompt medical intervention. Most of the reported cases involve young women, while HIV infection, pregnancy, and autoimmune diseases are the most common underlying conditions. Whether this condition can be triggered by the inactivated COVID-19 vaccine is unclear.

## Data Availability

The datasets obtained and/or analyzed during the current study are available from the corresponding author upon reasonable request.
